# Micro- and macro-changes in early-stage type 2 diabetes mellitus without cognitive impairment: a diffusion tensor imaging (DTI) and surface-based morphometry (SBM) study

**DOI:** 10.3389/fneur.2023.1115634

**Published:** 2023-07-05

**Authors:** Xiangyong Tang, Yanzi Chen, Hui Tan, Jinzhi Fang, Dafei Yu, Cuimei Chen, Xiao Li, Ziqi Hu, Ling Ding, Yuzhong Zhang

**Affiliations:** ^1^Department of Medical Imaging, Affiliated Longhua People's Hospital, Southern Medical University, Shenzhen, China; ^2^Department of Medical Imaging, Second Affiliated Hospital, Shantou University Medical College, Shantou, China

**Keywords:** type 2 diabetes mellitus, white matter, cortex, diffusion tenser imaging, FreeSurfer

## Abstract

**Introduction:**

Brain structure and function changes are considered major brain damages in type 2 diabetes mellitus (T2DM), which likely has a close relationship with cognitive impairment. Many previous studies have shown by using brain structural and functional magnetic resonance imaging (MRI) methods that brain white and gray matter are damaged in T2DM, leading to cognitive impairment. Researches neglected patients of T2DM without cognitive dysfunction might also have brain changes.

**Methods:**

In this study, subjects with early stage T2DM with no cognitive dysfunction were enrolled to detect brain damages using the tract-based spatial statistics analysis (TBSS) method to demonstrate white matter (WM) micro changes and surface-based morphometry (SBM) method to assess cerebral cortex macro changes.

**Results:**

The whole-brain TBSS analysis revealed that there were no statistically significant changes in fractional anisotropy (FA) and mean diffusivity (MD), but the FA declined in some area of cerebral WM (*p* < 0.1). The SBM results showed no changes in cortical thickness (CT), cortical volume (CV), surface area (SA), and cortical sulcal curve (CSC) between these two groups, but pial local gyration index (LGI) was decreased in the precuneus (−log10, *p* = −3.327).

**Discussion:**

In conclusion, early stage T2DM patients without cognitive impairment had brain micro and macro structural damages, suggesting the potential use of MRI as an imaging marker to detect brain changes in early stage T2DM, which could not be observed and assessed clinically.

## 1. Introduction

Type 2 diabetes mellitus (T2DM) is a common chronic metabolic disease with an increasing worldwide prevalence, and is known to involve many organ systems in the human body (1, 2). The number of patients with T2DM may increase to 42.3 million by the year 2030 (3). This condition is widely accepted as a risk factor for neurological disorders such as impairments to visual construction, planning, visual memory, and cognitive speed (4), all of which can easily lead to mild cognitive impairment (MCI), Alzheimer's disease, and dementia (5, 6). A number of neuroimaging studies have shown that structural abnormalities in the gray matter (GM) and white matter (WM) may account for cognitive disorders in patients with T2DM (7–9). These studies have not only focused on the differences between T2DM patients and healthy controls in terms of changes in WM microstructure (7, 8), GM volume losses (9), and cerebral cortex damage (10), but have also compared T2DM patients with and without cognitive impairment (11–13). However, the type of cognitive disorder caused by T2DM leads to clinical complications over a long period. The possibility that early-stage T2DM patients without cognitive impairment may also have brain structural damage has been neglected clinically, and this damage might subsequently have an impact on cognitive function or aggravate the progression of cognitive decline. This study aimed to detect micro-alterations in the WM and the macro-cortical structure in early-stage T2DM patients without cognitive dysfunction or complication with diabetes encephalopathy.

Magnetic resonance imaging (MRI) is a non-invasive way to explore structural changes within the brains of patients with diabetes. Previous studies have demonstrated that diffusion tensor imaging (DTI) is another non-invasive live imaging technique that can be used to detect the diffusion properties of water molecules in different cerebral regions, thereby providing information on the orientation of WM fiber bundles (14). These changes cannot be easily captured by conventional MRI techniques such as T1-weighted or T2-weighted imaging (T1WI and T2WI). However, three dimensional-T1WI, as a high-resolution imaging technique, can provide unambiguous and precise pictures of the brain, which can offer detailed information through post-processing techniques such as surface-based morphometry (SBM) (15) and voxel-based morphometry (VBM) (15). SBM is widely used to calculate a variety of features of the cortex, such as cortical thickness (CT), cortical volume (CV), surface area (SA), cortical sulcal curve (CSC), and pial local gyration index (LGI) (15, 16), while the VBM method can only serve to provide an estimate of GM volume (16). Hence, the aim of the present study was to evaluate patients with early-stage T2DM using the DTI and SBM methods in order to investigate changes to the WM fiber bundles and structure of the cortex.

## 2. Materials and methods

### 2.1. Participants

All participants in this study were recruited from outpatient or inpatient clinics of Affiliated Longhua People's Hospital, Southern Medical University between December 2021 and July 2022. Diagnosis of T2DM was based on established criteria according to the recommendations of the American Diabetes Association (diabetes symptoms and fasting plasma glucose >7.0 mmol/L, or random plasma glucose >11.1 mmol/L, or 2-h glucose >11.1 mmol/L after an oral glucose tolerance test) (17). A tract-based spatial statistics (TBSS) DTI study of WM abnormalities and a FreeSurfer-based SBM study of cortical structure were performed with 29 T2DM patients (patient group [PG]: 23 men and 6 women; mean age: 41.62 years, standard deviation [SD]: 1.93) and 25 healthy controls (HCs) matched on age, sex, education, and body mass index (BMI) (17 men and 8 women; mean age: 37.04 years, SD: 1.72). All T2DM patients had no cerebral complications or complications associated with other organs. All participants were right-handed and underwent two widely used cognitive tests, namely the Mini-Mental State Examination (MMSE) and the Montreal Cognitive Assessment (MoCA). Subjects who scored more than 26 (≥27) on the MMSE and more than 25 (≥26) on the MoCA were recruited for participation in this study. The exclusion criteria for participation in this study were as follows: (1) older than 60 years, with a course of disease >10 years; (2) history of psychological and/or neurological disorders or brain trauma or infection; (3) WM hyperintensity, age-related white matter change (ARWMC) score ≥2, >5 lesions; (4) history of cerebral ischemia and hypoxia; (5) hypertension, hyperlipemia, thyroid dysfunction, liver and kidney failure, severe anemia, or rheumatic immune system diseases; and (6) alcoholism, smoking addiction, long-term drug use, or drug dependence. None of the participants had any obvious contraindication for MRI. Table 1 presents the characteristics of participants included in the study.

**Table 1:** Subject characteristics.

**Characteristic**	**PG**	**HCs**	**χ^2^/t/z**	***p*-value**
Male/Female	23 (6)	17 (8)	2.07	0.15
Age (years)	41.62 ± 1.93	37.04 ± 1.72	−1.55	0.13
SBP (mmHg)	116.08 ± 2.86	114.08 ± 2.13	0.48	0.91
DBP (mmHg)	72.76 ± 1.71	70.60 ± 2.13	0.11	0.86
BMI	22.53 ± 0.51	22.59 ± 0.28	−0.12	0.9
Education (years)	13.00 (10.00, 16.00)	12.00 (10.5, 16.00)	−1.14	0.26
Course (years)	4 (2, 7)	N/A	N/A	N/A
FBG (mmol/L)	10.88 ± 0.81	4.57 ± 0.17	7.13	0.00^*^
HbA1c (%)	9.77 ± 2.86	5.35 ± 0.11	7.59	0.00^*^
MMSE	28.34 ± 1.11	28.76 ± 1.16	0.124	0.73
MoCA	28.21 ± 1.35	28.24 ± 1.30	0.01	0.98
Right-handed	100%	100%		

Data are expressed in the form of mean ± standard deviation (SD) or median (quartiles) according to whether the variabled conformed to the normal distribution. Sex was compared using the chi-square test (cross-analysis). Age, SBP, DBP, BMI, FBG, HbA1c, MMSE, and MoCA were compared using independent samples *t*-tests. Education and course years were analyzed using a non-parametric test. PG, patient group; HCs, healthy controls; SBP, systolic blood pressure; DBP, diastolic blood pressure; BMI, body mass index; FBG, fasting blood glucose; MMSE, Mini-Mental State Examination; MoCA, Montreal Cognitive Assessment. N/A indicates no relevant data. ^*^p < 0.01.

### 2.2. MRI acquisition

All MRI data, including DTI and 3D-T1WI images, were acquired on a 3.0T MR Scanner (Siemens) equipped with a standard 16-channel phased-array head coil. We acquired DTI images using the spin echo phase scan. The parameters of the DTI sequence were as follows: flip angle = 90°, echo time (TE) = 89 ms, repetition time (TR) = 9,240 ms, field of view (FOV) = 224 mm × 224 mm, thickness = 2 mm, slices = 75, gap = 0 mm, acquisition voxel size = 2 mm × 2 mm × 2 mm, b-value = 1,000 s/mm^2^ with an additional b0 (b-value = 0) image, sequence scan time = 6 min 30 s. Next, we used a sagittal T1-weighted 3D brain sequence to obtain high-resolution anatomical images with the following parameters: TR = 600 ms, TE = 28 ms, FOV = 250 mm × 250 mm, matrix = 252 × 250, voxel size = 1 mm × 1 mm × 1 mm, gap = −0.5 mm, number of signal averages (NSA) = 1, flip angle = 90°. We also obtained T2WI and T2-weighted fluid-attenuated inversion recovery (T2-FLAIR) images. The T2WI scan parameters were TR = 3,000 ms, TE = 105 ms, thickness = 5 mm; the T2-FLAIR parameters were TR = 9,000 ms, TE = 130 ms, inversion time (TI) = 2,500 ms, thickness = 5 mm.

### 2.3. TBSS data analysis

The DTI data analysis was based on TBSS in our study. All DTI data were initially converted to Neuroimaging Informatics Technology Initiative (NIFTI) format. The data quality was checked, and subjects with poor data quality and obvious head movement were excluded. As described in a previous TBSS study (4), the following DTI data processing methods were employed to analyze the diffusion characteristics of the WM using the Functional MRI of the Brain (FMRIB) Software Library (FSL) (www.fmrib.ox.ac.uk/fsl), which was developed by Oxford University. We first utilized the FMRIB Diffusion Toolbox (FDT) 3.0 for simultaneous correction of eddy-current distortions and rigid-body head motion. We corrected the gradient direction and utilized the Brain Extraction Tool (BET) based on the b0 image to subsequently create a brain mask. Finally, we used the dtifit function to calculate and obtain images for the fractional anisotropy (FA) and mean diffusivity (MD) values (https://fsl.fmrib.ox.ac.uk/fsl/fslwiki/TBSS/UserGuide).

After the above-described post-processing procedure, voxel-wise statistical analysis was performed with TBSS to evaluate intergroup differences in WM (18). We created an FA file and used the command tbss_1_preproc ^*^.nii.gz command to check the FA images. Next, the command tbss_2_reg –T/-t/-n command was used to register FA to the FMRIB 58_FA template by linear and non-linear registration methods. Subsequently, the command tbss_3_postreg –S/-T command was used to build the mean FA image and mean FA skeleton. The command tbss_4_prestats 0.2 command was then used to take a 0.2 threshold for the mean FA skeleton. Finally, the command design_ttest2 design N1 N2 was run, wherein N1 and N2 represented the labels of each of the two groups of subjects. Statistical analysis was based on permutation tests with randomization command (5,000 times) and multiple comparison correction by threshold-free cluster enhancement (TFCE) command.

### 2.4. SBM data analysis

The T1 images were processed using FreeSurfer 6.0.0 (http://surfer.nmr.mgh.harvard.edu, Harvard University, USA) with a series of automatic sequences to reconstruct the cortical surface and cortical thickness of the whole brain based on the template-driven approach, as described previously (19, 20) for volume and surface segmentation. Briefly, the entire process was as follows: motion correction; removal of non-brain tissue; Talairach transform computation; intensity normalization; topology correction, segmentation of subcortical structures; creation of the borders of the pial surface and surface of the WM and GM; surface inflation; segmentation of the cerebral cortex into units based on the gyrus and sulcus structure; and creation of a variety of surface-based data. The definition of the cortical thickness at each point was taken to be the shortest distance between the pial surface and the WM and GM border. All of the corrected cortical thickness maps were used to generate a common average surface and were smoothed using a Gaussian kernel of 10 mm full-width half-maximum; pial LGI was smoothed using a Gaussian kernel of 5 mm full-width half-maximum. The LGI parameter was used to calculate the local gyration indices of each subject using Matlab2010a.

### 2.5. Statistical analysis

A general linear model (GLM) tool was used to conduct two-sample *t*-tests to compare the PG and HCs, with age, sex, and education after mean removal treated as covariates. A permutation test was used to conduct statistical analysis for each voxel on the FA skeleton, with the number of permutations set to 5,000. Multiple comparison correction was performed using the threshold-free cluster enhancement (TFCE) method, which is similar to the cluster-based correction method but is generally more reliable and avoids the use of arbitrary initial cluster-threshold values. A corrected *p*-value of 0.05 was used as the significance threshold. Brain region information for fiber tracts was identified according to the Johns Hopkins University (JHU) White-matter Tractography Atlas and JHU International Consortium for Brain Mapping (ICBM)-DTI-81 White-matter Labels for significantly different masses.

Differences in CT, CV, CSC, SA, and LGI between the PG and HCs were examined using two-sample *t*-tests with age, sex, and education level as covariates. The absolute value of the vertex threshold was 2, and the cluster-level Monte Carlo simulation method was used to adjust for multiple comparisons. Vertex *p* < 0.01 and cluster *p* < 0.05 were used as the significance threshold.

## 3. Results

No specific lesions were found in any of the subjects on the conventional MRI scans.

### 3.1. TBSS results of WM

**Figure 1:**
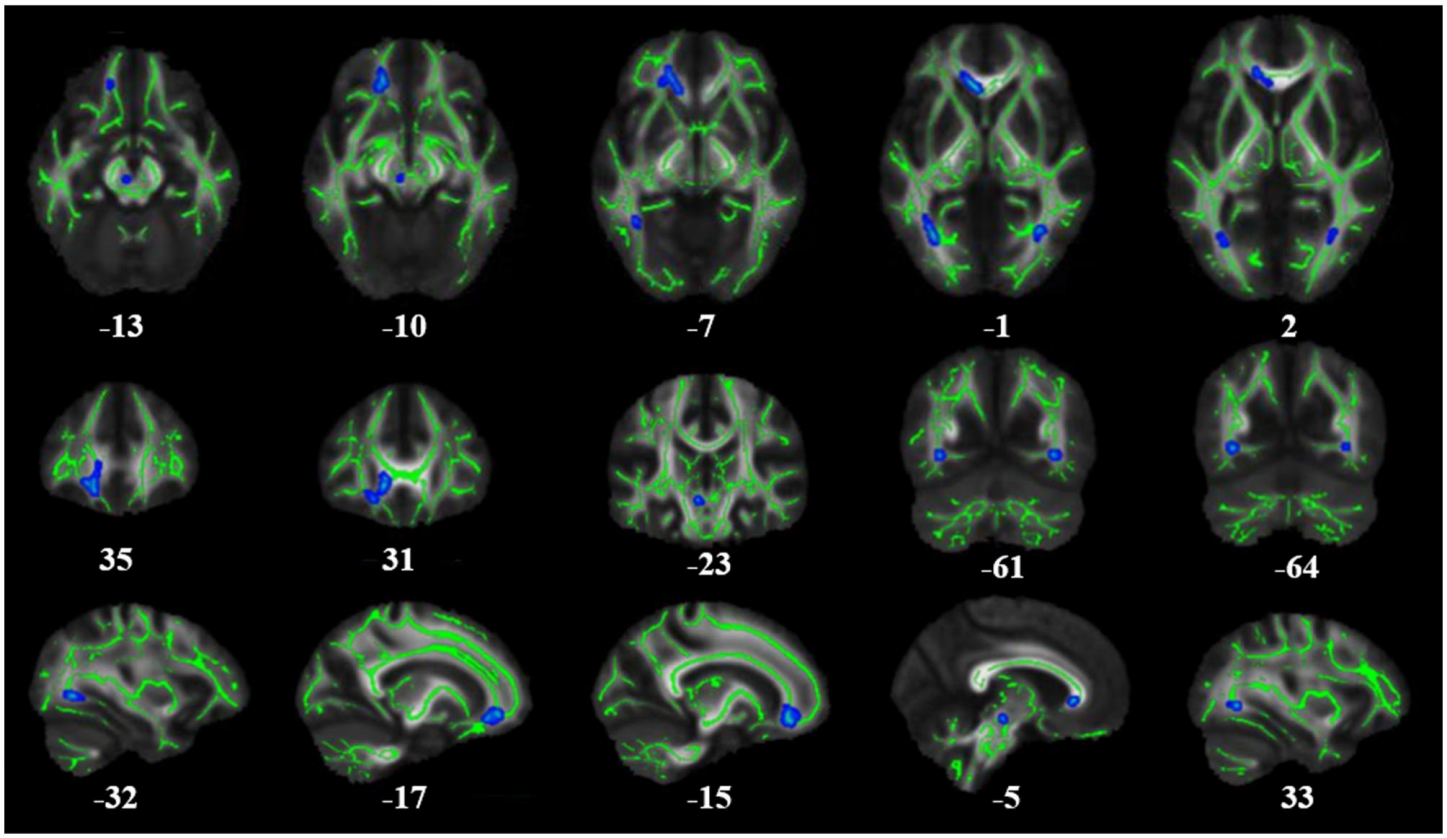
Results of whole-brain tract-based spatial statistics comparing FA in patients with T2DM and healthy controls (HCs). The images show significantly decreased FA values in T2DM patients compared to HCs. The skeleton of the white matter fiber bundle is shown in green, and the area with decreased FA value is indicated in blue. FA, fractional anisotropy.

There was no significant difference between the PG and HCs in the TBSS value of WM. We also found no statistically significant differences in FA or MD between patients with early-stage T2DM without cognitive impairment and HCs (*p* < 0.05). Figure 1 and Table 2 show the TBSS results of the direct comparison. Analyses of the T2DM data revealed a trend toward decrease of FA values in these patients. At a threshold of *p* < 0.1, the FA value of several brain areas was found to be reduced in T2DM, namely, the genu of the corpus callosum, forceps minor, left and right posterior thalamic radiation, and left and right inferior fronto-occipital fasciculus (*p* < 0.1) (Table 2).

**Table 2:** Brain regions with changes in FA (*p* < 0.1) and their location and size.

**Encephalic regions**	**X**	**Y**	**Z**	***T*-value**	**Corrected *p*-value**	**Mass size**
Genu of corpus callosum, forceps minor	−15	35	−12	3.11	0.091	197
Posterior thalamic radiation (include optic radiation) L, inferior fronto-occipital fasciculus L	−32	−64	0	4.60	0.089	88
Anterior corona radiata L, uncinate fasciculus L	−17	31	−10	2.44	0.098	46
Posterior thalamic radiation (include optic radiation) R, inferior fronto-occipital fasciculus R	33	−61	0	4.53	0.096	30
Anterior thalamic radiation L	−5	−23	−12	4.65	0.096	19

### 3.2. SBM results for the cerebral cortex

**Figure 2:**
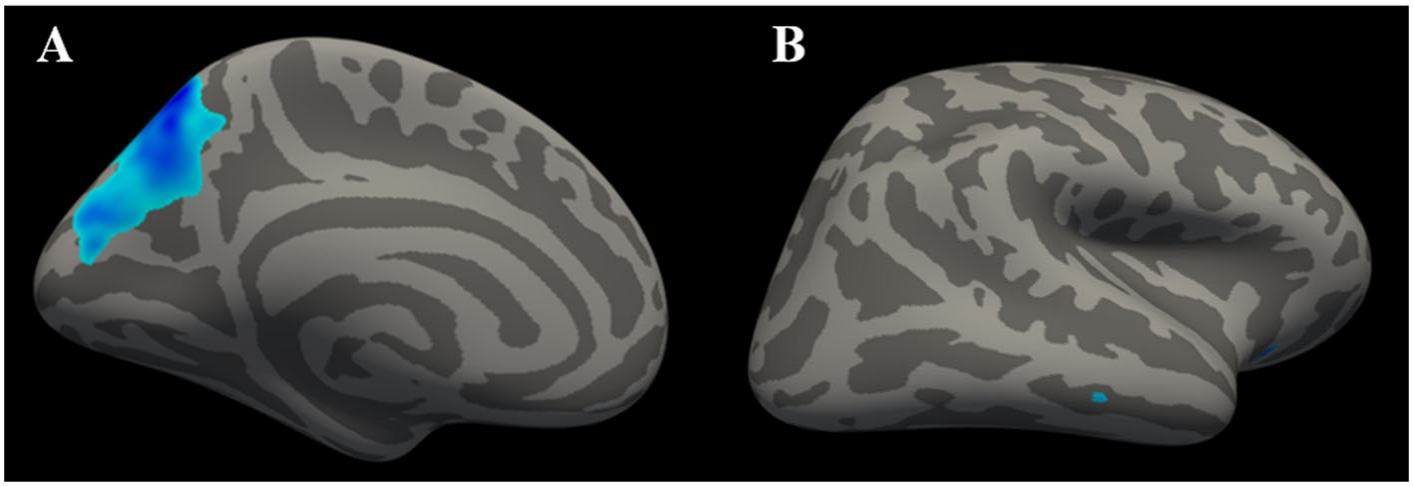
Clusters with significant inter-group differences in LGI, corrected for age, sex, and education (Monte Carlo simulation method). **(A)** shows the left cerebral hemisphere (vertex *p* < 0.01, cluster *p* < 0.05), indicating that the LGI of the precuneus was significantly lower in T2DM patients than in healthy controls. **(B)** shows the right cerebral hemisphere (vertex *p* < 0.01, cluster *p* > 0.05), with the absence of significant difference in LGI on the right side of the brain.

According to the SBM analysis conducted using FreeSurfer, there were no statistically significant differences between the patients with early-stage T2DM and HCs in terms of the cerebral cortex parameters of CT, CV, SA, and CSC (vertex *p* < 0.01, cluster *p* >0.05). However, comparison of the cortical parameter of pial LGI showed that there was a significant difference between the groups in the left precuneus. Compared with HCs, subjects with early-stage T2DM might exhibit a decline of pial LGI in the left precuneus (vertex *p* < 0.01, cluster *p* < 0.05). As shown in Table 3 and Figure 2A, there was a significant difference between the groups in the left precuneus of pial LGI. As shown in Table 4 and Figure 2B, no difference was observed in the right cerebral hemisphere.

**Table 3:** Inter-group difference in pial LGI in the left cerebral hemisphere.

**Encephalic region**	**MNI peak coordinates**			**-log_10_ *p*-value**	**Cluster size (mm^2^)**
	**X**	**Y**	**Z**		
Precuneus	−7.6	−71.3	51.1	−3.372	46,871

Vertex *p* < 0.01, cluster *p* < 0.05. A -log10 *p*-value < 0 indicates that the mass LGI was lower in T2DM patients than in healthy controls. MNI, Montreal Neurological Institute.

**Table 4:** Inter-group differences in pial LGI in the right cerebral hemisphere.

**Encephalic region**	**MNI peak coordinates**			**–log10 *p*-value**	**Cluster size (mm^2^)**
	**X**	**Y**	**Z**		
Lateral orbitofrontal	28.0	19.3	−19.6	−2.318	23.37
Middle temporal	54.6	−27.3	−18.2	−2.052	13.56
Superior parietal	10.7	−72.9	50.2	−2.026	4.02

Vertex *p* < 0.01, cluster *p* > 0.05. A -log10 *p*-value < 0 indicates that the mass LGI was lower in T2DM patients than in healthy controls. MNI, Montreal Neurological Institute.

## 4. Discussion

T2DM, as a common metabolic disease, can influence the function of multiple organs and organ systems. Brain WM and GM are among the target tissues for diabetic organ damage. The changes in brain structure and function caused by T2DM are indistinct; hence, this has created significant research interest among many scholars. In this study, changes in WM microarchitecture in patients with early-stage T2DM were analyzed through TBSS analysis of DTI, and changes in cerebral cortex macrostructure were analyzed via the SBM method using the FreeSurfer software package. Any such changes might be closely related to cognitive function, leading to a high risk of Alzheimer's disease and dementia among T2DM patients. Therefore, it is necessary to study the relationship between diabetes and damage to cognitive function. We first evaluated the cognitive function of all participants using the internationally recognized assessment methods of the MMSE and MoCA. We recruited patients with early-stage T2DM without cognitive impairment, in contrast with many previous studies, which have either enrolled T2DM patients with cognitive impairment or not distinguished between the two groups based on cognitive function. Furthermore, some studies have even selected patients with T2DM with complications of various systems. Few studies have recruited cognitively normal early-stage T2DM patients as their patient population. However, in the present study, early-stage T2DM patients with clinically normal cognitive function did not show any micro-changes in their brain tissues. Our study was devoted to understanding changes in brain WM and in the cortex in early-stage T2DM.

In the DTI investigation, we analyzed data in combination with the TBSS method to investigate WM abnormalities. We found that there were no obvious changes in FA values in early-stage T2DM patients without cognitive impairment. Nevertheless, there was a trend toward a decrease in FA in the following regions: the genu of the corpus callosum, forceps minor, left and right posterior thalamic radiation, and left and right inferior fronto-occipital fasciculus (*p* < 0.1). This means although we did not find significant changes in the brain WM microstructure in early-stage T2DM patients, a trend toward decrease in FA might occur, indicating the possibility of potential ongoing damage to the WM as the hyperglycemic state continues, with potential loss of the integrity of WM fiber bundles. These differences were observed in regions of WM strongly associated with cognitive impairment, and the damage to these regions might become more apparent as the disease progresses. Many previous studies of T2DM with cognitive impairment have also found changes in WM brain structure. Xiong et al. (1) demonstrated decreased FA and increased MD in patients with MCI compared with normal controls using whole-brain TBSS analysis. Tan et al. also found decreased FA and increased MD and axial diffusivity (AD) in several WM regions and tracts among T2DM patients as compared to HCs (4). A meta-analysis conducted by Huang et al. identified 10 WM regions that showed a consistent reduction in FA in patients with T2DM (21). Decreased FA has been recognized by scholars as occurring in T2DM. However, insufficient attention has been paid to the distinction between T2DM patients with and without cognitive impairment in many previous studies, so these results have been based on a mixture of both of these groups of patients. In our study, patients with T2DM at an early stage and with no cognitive impairment were selected for recruitment, and the results only showed a trend toward a decrease in FA. These findings are mainly attributable to the fact that all our patients had early-stage T2DM, so there were no statistically significant differences (at a threshold of *p* < 0.05). Gradual, slow, and continuous WM change and the associated cognitive decline in patients with T2DM could still exist and could be captured by DTI parameters. These can also be used to complement neuropsychological test scores in identifying patients with T2DM with and without mild cognitive impairment in the future, and can also be used to help in timely intervention of the progress of diabetes encephalopathy.

We used MRI technology to further establish the neural structural basis for this study. In our SBM investigation, we analyzed the CT, CV, SA, and pial LGI parameters; we observed changes in pial LGI, but no changes in CT, CV, or SA. The results of the present study indicated that the LGI value for the left precuneus was lower in individuals with early-stage T2DM than in HCs. LGI is an indicator that can be used to investigate abnormal cortical folding (22). The precuneus constitutes the posterior region of the medial parietal cortex, which is an important area closely associated with cognitive processing (23). With widespread connectivity with both cortical and subcortical structures, the precuneus is functionally involved in diverse roles (23, 24). It plays a central role in a wide spectrum of highly integrated tasks, including visuospatial imagery, episodic memory retrieval, and self-processing operations (25, 26). Furthermore, using resting-state analysis, researchers have determined that the precuneus plays a core role not only in the default mode network but also more broadly through its engagement under a variety of processing states (26). In this study, the LGI of the precuneus was found to be reduced in early-stage T2DM, representing a series of anatomic and potential behavior changes, but these patients did not have any clinical symptoms. This change in precuneus LGI might be the earliest cerebral lesion and could be a potential indicator region for early-stage T2DM-related brain damage.

There was no significant difference in the parameters of CT, CV, or SA between early-stage T2DM patients and HCs; these results indicated that abnormal cortical changes were not clearly evident in individuals with T2DM with no cognitive impairment and at an early stage of the disease, with no clinical symptoms. Any morphometric change in the cortex was not obvious. Previous studies has shown that there are many risk factors for loss of cortical thickness and volume, such as age and blood pressure (27, 28). A study on chimpanzees demonstrated that as chimpanzees age, they lose GM in regions associated with cognition (27). Another study found that people with hypertension exhibit severe transformation of the brain structure (28). The patients who participated in our study were relatively young (mean age: 41.62 years), with no hypertension and/or any other systemic disease; hence, the influence of other factors on cortical changes could be excluded. For this reason, the results are relatively reliable and might provide some basic information on the clinical progress of diabetes encephalopathy.

Our study has several limitations. First, this was a cross-sectional study rather than a longitudinal study. We only recruited patients with early-stage T2DM and excluded those with advanced T2DM or with accompanying systemic complications. It would be useful to contrast these two subject groups to demonstrate any difference in cerebral WM and GM. Second, many patients with T2DM receive medication, and we neglected the influence of drugs on the central nervous system. We cannot rule out the possibility that our results partly reflect the effects of medication.

## 5. Conclusion

In this study, we used DTI to assess microstructural changes to WM in patients with early-stage T2DM with no cognitive impairment; the results showed that there were no significant changes in FA and MD, but there was a trend toward decreasing FA (*p* < 0.1). This trend could indicate the possibility of WM fiber bundle damage in early-stage T2DM. Analysis of macro structural changes in the cerebral cortex showed no difference between patients and HCs in CT, CV, or SA; however, there was a decline in LGI of the precuneus among T2DM patients, indicating that LGI may likely reflect early damage to the brain in T2DM, and the precuneus might be the early cerebral target of diabetes-related damage. To our knowledge, this is the first study to combine TBSS and SBM to investigate the early stage of T2DM without cognitive impairment. We believe that our results offer helpful information on macro and micro cerebral changes in T2DM, and these might act as a reference and aid in the understanding of the progress of diabetes encephalopathy and timely interventions that can be made.

## Data availability statement

The original contributions presented in the study are included in the article/Supplementary material, further inquiries can be directed to the corresponding author.

## Ethics statement

The studies involving human participants were reviewed and approved by Medical Ethics Committee of the Affiliated Longhua People's Hospital, Southern Medical University. The patients/participants provided their written informed consent to participate in this study.

## Author contributions

YZ was responsible for the project administration and contributed to the study design. XT carried out the data processing, statistical analysis, and wrote and edited the original draft. YC and HT helped with collection of the experimental data. JF, DY, CC, and XL conceived and designed the study. ZH and LD edited the manuscript with substantial input from XT. All authors read and approved the final manuscript.
