# Supplementary material for: Micro- and macro-changes in early-stage type 2 diabetes mellitus without cognitive impairment: a diffusion tensor imaging (DTI) and surface-based morphometry (SBM) study
Source: Front Neurol. 2023 Jul 5;14:1115634. doi: 10.3389/fneur.2023.1115634 (PMC10354865; doi:10.3389/fneur.2023.1115634)
Supplement: Supplementary file 1 [file Data_Sheet_1.docx]

Supplementary Material

**Micro and macro changes in early stage of type 2 diabetes mellitus without cognitive impairment: a DTI and SBM study**

**Xiangyong Tang^1^, Yanzi Chen^1^, Hui Tan^2^, Jinzhi Fang^1^, Dafei Yu^1^, Cuimei Chen^1^, Xiao Li ^1^, Ziqi Hu^1^, Ling Ding^1^ and Yuzhong Zhang^1*^**

*** Correspondence:** Corresponding Author: Yuzhong Zhang, email: 751948406@qq.com

#

# Supplementary Figures and Tables

Table 1 shows the change regions parameters of cortical thickness (CT) in left brain (vertex *p* < 0.01, cluster *p* > 0.05). These parameters are MNI peak coordinates, -log10*p* value and size of them. -log10 *p* value < 0 means the mass CT in T2DM patients was lower than that of health controls. -log10 *p* value > 0 is the opposite.

| Encephalic regions | MNI peak coordinates | | | -log10*p* value | | Cluster Size (mm^2^) |
| --- | --- | --- | --- | --- | --- | --- |
|  | X | Y | Z | |  |  |
| inferiorparietal | -42 | -64.2 | 14.9 | | -2.498 | 50.80 |
| superiortemporal | -56.8 | -30.5 | 6.1 | | -2.166 | 12.20 |
| superiorparietal | -34.8 | -53.2 | 58.1 | | 2.075 | 9.01 |
| entorhinal | -20.6 | -9 | -30.7 | | -2.313 | 8.83 |

Table 2 shows the change regions parameters of CT in right brain (vertex *p* < 0.01, cluster *p* > 0.05). These parameters are MNI peak coordinates, -log10*p* value and size of them. -log10 *p* value < 0 means the mass CT in T2DM patients was lower than that of health controls. -log10 *p* value > 0 is the opposite.

| Encephalic regions | MNI peak coordinates | | | -log_10_*p* value | Cluster Size (mm^2^) |
| --- | --- | --- | --- | --- | --- |
|  | X | Y | Z |  |  |
| fusiform | 40.7 | -22.4 | -21.8 | -2.959 | 62.9 |
| superiorparietal | 19.6 | -57.7 | 63.4 | -2.411 | 29.49 |
| superiorparietal | 11.8 | -49.7 | 70.1 | 2.598 | 22.15 |
| superiortemporal | 49.7 | -16.2 | -1.3 | -2.137 | 19.14 |
| postcentral | 46.2 | -24.5 | 48.1 | 2.087 | 5.44 |

Table 3 shows the change regions parameters of surface area (SA) in left brain (vertex *p* < 0.01, cluster *p* > 0.05). These parameters are MNI peak coordinates, -log10*p* value and size of them. -log10 *p* value < 0 means the mass SA in T2DM patients was lower than that of health controls. -log10 *p* value > 0 is the opposite.

| Encephalic regions | MNI peak coordinates | | | -log_10_*p* value | Cluster Size (mm^2^) |
| --- | --- | --- | --- | --- | --- |
|  | X | Y | Z |  |  |
| fusiform | -37 | -40.8 | -22.6 | -2.905 | 483.38 |
| precuneus | -10.9 | -64.2 | 23 | -2.127 | 47.79 |
| precuneus | -18.6 | -72.5 | 29.6 | -2.081 | 30.6 |
| postcentral | -37.7 | -30.2 | 60.7 | -2.124 | 23.19 |
| precentral | -53.5 | -3 | 40.9 | -2.007 | 0.93 |

Table 4 shows the change regions parameters of SA in right brain (vertex *p* < 0.01, cluster *p* > 0.05). These parameters are MNI peak coordinates, -log10*p* value and size of them. -log10 *p* value < 0 means the mass SA in T2DM patients was lower than that of health controls. -log10 *p* value > 0 is the opposite.

| Encephalic regions | MNI peak coordinates | | | -log_10_*P* value | Cluster Size (mm^2^) |
| --- | --- | --- | --- | --- | --- |
|  | X | Y | Z |  |  |
| temporalpole | 36.7 | 4.1 | -28.3 | -3.448 | 199.49 |
| superiorfrontal | 10.8 | 53.2 | 8.9 | -3.464 | 158.53 |
| precentral | 35.9 | -15.7 | 35.9 | -2.659 | 47.64 |
| superiortemporal | 44.1 | -32.8 | -2.3 | -2.411 | 44.02 |
| postcentral | 63.7 | -8.6 | 23.0 | -2.142 | 11.75 |
| inferiorparietal | 38.4 | -82.3 | 20.0 | -2.050 | 10.07 |
| precentral | 45.0 | -8.8 | 36.7 | -2.034 | 4.11 |
| insula | 37.0 | 2.0 | -20.5 | -2.297 | 0.33 |

Table 5 shows the change regions parameters of cortical volume (CV) in left brain (vertex *p* < 0.01, cluster *p* > 0.05). These parameters are MNI peak coordinates, -log10*p* value and size of them. -log10 *p* value < 0 means the mass CV in T2DM patients was lower than that of health controls. -log10 *p* value > 0 is the opposite.

| Encephalic regions | MNI peak coordinates | | | -log_10_*p* value | Cluster Size (mm^2^) |
| --- | --- | --- | --- | --- | --- |
|  | X | Y | Z |  |  |
| medialorbitofrontal | -3.6 | 36.7 | 23.2 | -2.593 | 109.32 |
| precentral | -39.0 | -1.6 | 16.6 | -2.441 | 105.08 |
| fusiform | -41.5 | -31.5 | 21.2 | -2.187 | 55.51 |
| postcentral | -49.0 | -19.1 | 19.0 | -2.214 | 41.73 |
| parsopercularis | -42.7 | 15.9 | 7.8 | -2.337 | 39.63 |
| superiortemporal | -54.4 | 0.5 | -7.6 | -2.321 | 37.38 |
| precuneus | -18.8 | -71.7 | 28.7 | -2.339 | 36.61 |
| supramarginal | -59.0 | -28.6 | 19.0 | -2.130 | 30.86 |
| superiortemporal | -49.8 | -27.0 | -4.1 | -2.331 | 26.96 |
| insula | -34.2 | 3.9 | 3.6 | -2.017 | 2.17 |

Table 6 shows the change regions parameters of CV in right brain (vertex *p* < 0.01, cluster *p* > 0.05). These parameters are MNI peak coordinates, -log10*p* value and size of them. -log10 *p* value < 0 means the mass CV in T2DM patients was lower than that of health controls. -log10 *p* value > 0 is the opposite.

| Encephalic regions | MNI peak coordinates | | | -log_10_*p* value | Cluster Size (mm^2^) |
| --- | --- | --- | --- | --- | --- |
|  | X | Y | Z |  |  |
| superiorfrontal | 14.6 | 46.7 | 9.0 | -4.153 | 212.69 |
| superiortemporal | 43.4 | -0.8 | -21.6 | -2.601 | 112.15 |
| insula | 36.4 | -9.3 | -8.9 | -2.755 | 100.46 |
| superiortemporal | 46.7 | -20.1 | -0.0 | -2.892 | 78.54 |
| inferiorparietal | 35.9 | -81.6 | 20.6 | -2.332 | 58.77 |
| superiorparietal | 12.2 | -49.7 | 70.1 | 2.699 | 56.94 |
| bankssts | 44.5 | -40.2 | 0.8 | -2.543 | 52.73 |
| superiorparietal | 19.3 | -56.8 | 62.5 | -2.783 | 47.53 |
| supramarginal | 44.3 | -32.7 | 26.1 | -2.487 | 46.93 |
| inferiortemporal | 54.6 | -20.0 | -25.1 | -2.411 | 36.80 |
| superiorparietal | 20.0 | -81.0 | 41.5 | -2.230 | 35.15 |
| middletemporal | 53.7 | -31.0 | -10.1 | -2.116 | 25.90 |
| inferiortemporal | 56.3 | -55.5 | -12.2 | -2.116 | 16.20 |
| postcentral | 50.6 | -13.1 | 30.6 | -2.041 | 4.98 |
| precentral | 36.4 | 4.5 | 25.8 | -2.048 | 4.73 |

Table 7 shows the change regions parameters of cortical sulcal curve (CSC) in left brain (vertex *p* < 0.01, cluster *p* > 0.05). These parameters are MNI peak coordinates, -log10*p* value and size of them. -log10 *p* value < 0 means the mass CSC in T2DM patients was lower than that of health controls. -log10 *p* value > 0 is the opposite.

| Encephalic regions | MNI peak coordinates | | | -log_10_*p* value | Cluster Size (mm^2^) |
| --- | --- | --- | --- | --- | --- |
|  | X | Y | Z |  |  |
| inferiorparietal | -42.6 | -51.7 | 17.9 | -2.855 | 28.51 |
| fusiform | -31.1 | -46.2 | -15.1 | 2.803 | 28.08 |
| inferiortemporal | -54.3 | -23.3 | -28.8 | 3.057 | 27.62 |
| superiortemporal | -49.4 | -28.2 | -3.6 | 2.438 | 27.41 |
| pericalcarine | -7.7 | -81.5 | 12.0 | -2.413 | 23.19 |
| fusiform | -40.5 | -54.8 | -20.3 | -2.315 | 18.25 |
| parsopercularis | -54.1 | 18.0 | 16.4 | -2.631 | 17.88 |
| isthmuscingulate | -14.5 | -44.1 | -3.3 | -2.309 | 14.71 |
| inferiorparietal | -40.7 | -66.5 | 38.9 | -2.315 | 13.47 |
| inferiorparietal | -33.7 | -76.0 | 41.9 | 2.184 | 13.12 |
| superiortemporal | -52.5 | 5.0 | -11.6 | 2.451 | 12.54 |
| inferiorparietal | -41.9 | -63.9 | 14.7 | 2.122 | 10.41 |
| precentral | -35.7 | -18.7 | 65.2 | 2.331 | 7.19 |
| isthmuscingulate | -4.2 | -40.1 | 29.0 | 2.223 | 7.03 |
| caudalanteriorcingulate | -5.1 | 25.6 | 22.1 | 2.186 | 6.59 |
| lateraloccipital | -23.5 | -95.5 | -16.5 | 2.166 | 6.31 |
| precuneus | -19.1 | -60.3 | 12.2 | 2.092 | 3.66 |
| precuneus | -5.2 | -57.2 | 15.2 | 2.120 | 3.47 |
| superiorfrontal | -12.4 | -4.3 | 43.2 | -2.179 | 3.38 |
| precuneus | -5.8 | -62.2 | 29.2 | 2.166 | 3.22 |
| bankssts | -49.2 | -46.8 | 10.1 | 2.121 | 3.20 |
| precentral | -24.5 | -20.0 | 70.2 | -2.200 | 2.54 |
| parahippocampal | -26.5 | -24.0 | -26.8 | -2.017 | 0.89 |
| inferiorparietal | -50.8 | -58.4 | 30.8 | 2.003 | 0.54 |
| insula | -35.2 | 0.2 | -24.2 | -2.008 | 0.29 |

Table 8 shows the change regions parameters of cortical sulcal curve (CSC) in right brain (vertex *p* < 0.01, cluster *p* > 0.05). These parameters are MNI peak coordinates, -log10*p* value and size of them. -log10 *p* value < 0 means the mass CSC in T2DM patients was lower than that of health controls. -log10 *p* value > 0 is the opposite.

| Encephalic regions | MNI peak coordinates | | | -log_10_*p* value | Cluster Size (mm^2^) |
| --- | --- | --- | --- | --- | --- |
|  | X | Y | Z |  |  |
| superiorfrontal | 14.6 | 46.5 | 9.6 | 3.434 | 99.89 |
| rostralanteriorcingulate | 10.1 | 40.3 | 6.1 | -4.186 | 97.84 |
| fusiform | 41.9 | -29.6 | -20.3 | 2.375 | 72.34 |
| superiorparietal | 17.5 | -58.8 | 63.3 | 2.960 | 65.85 |
| superiortemporal | 46.3 | -31.3 | 8.8 | 3.658 | 65.79 |
| inferiorparietal | 47.1 | -51.0 | 41.6 | 2.771 | 62.65 |
| rostralmiddlefrontal | 21.4 | 56.8 | -9.6 | 3.465 | 41.96 |
| inferiortemporal | 55.9 | -53.1 | -12.6 | 2.848 | 41.68 |
| superiorparietal | 19.5 | -60.4 | 52.5 | -2.714 | 34.43 |
| inferiortemporal | 55.2 | -21.2 | -25.1 | 2.818 | 27.22 |
| supramarginal | 46.6 | -32.5 | 28.2 | 2.604 | 22.70 |
| superiorparietal | 29.9 | -39.6 | 45.6 | 2.769 | 22.68 |
| precuneus | 9.0 | -49.1 | 54.6 | -2.500 | 19.76 |
| precuneus | 9.7 | -52.1 | 47.3 | 2.461 | 16.86 |
| transversetemporal | 37.0 | -29.3 | 12.9 | -2.422 | 11.18 |
| insula | 35.0 | -18.9 | 11.7 | 2.150 | 7.62 |
| temporalpole | 33.3 | 13.7 | -34.9 | 2.252 | 6.82 |
| postcentral | 42.6 | -24.4 | 56.0 | 2.179 | 6.80 |
| postcentral | 17.1 | -31.5 | 73.8 | 2.230 | 4.79 |
| superiorparietal | 19.5 | -65.3 | 43.5 | 2.003 | 0.55 |

## Supplementary Figures


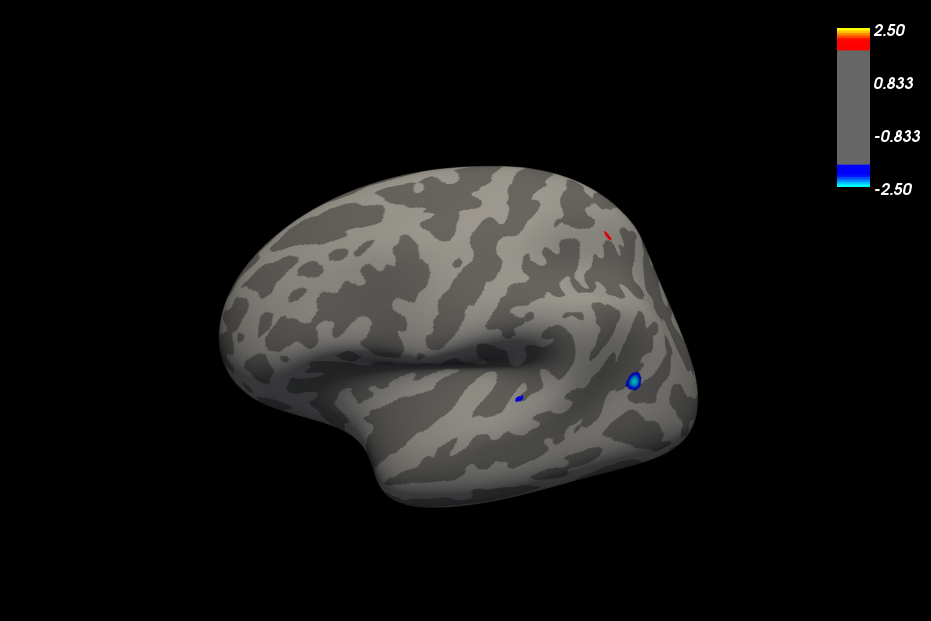


**Supplementary Figure 1.** Clusters with no significant group differences in CT analysis of left cerebrum. Regions with blue color represent the CT of these area was lower in T2DM than health controls, but these are not significantly difficult (vertex *p* < 0.01, cluster *p* > 0.05). Red regions are the opposite.


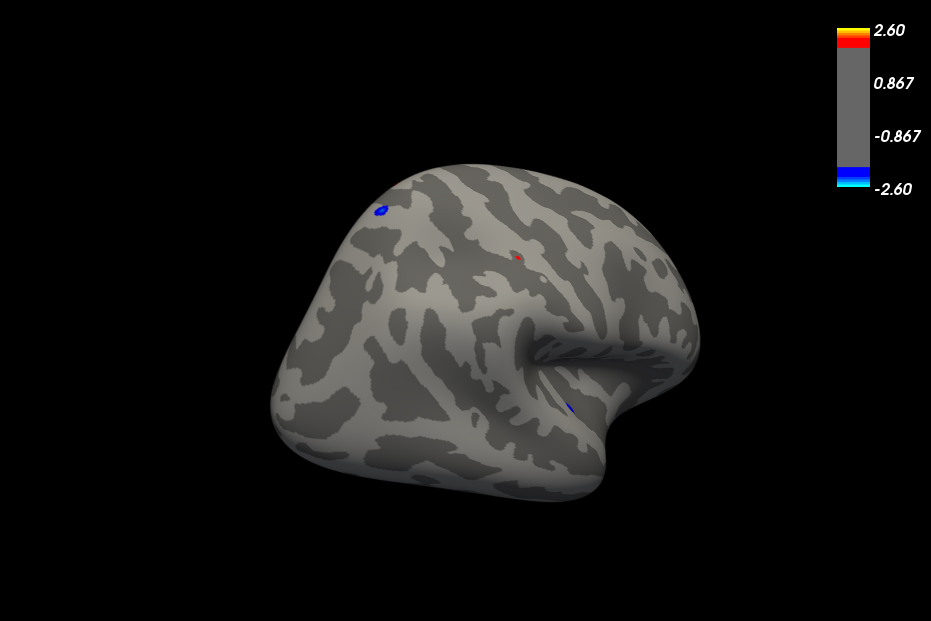


**Supplementary Figure 2.** Clusters with no significant group differences in CT analysis of right cerebrum. Regions with blue color represent the CT of these area was lower in T2DM than health controls, but these are not significantly difficult (vertex *p* < 0.01, cluster *p* > 0.05). Red regions are the opposite.


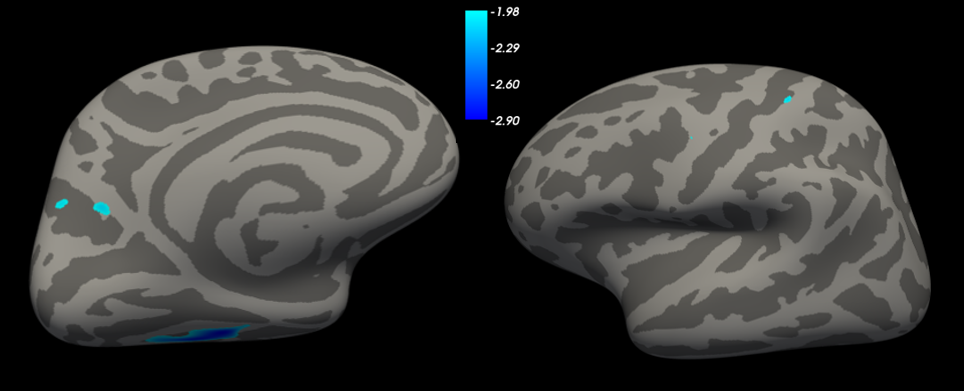


**Supplementary Figure 3.** Clusters with no significant group differences in SA analysis of left cerebrum. Regions with blue color represent the SA of these area was lower in T2DM than health controls, but these are not significantly difficult (vertex *p* < 0.01, cluster *p* > 0.05). Red regions are the opposite.


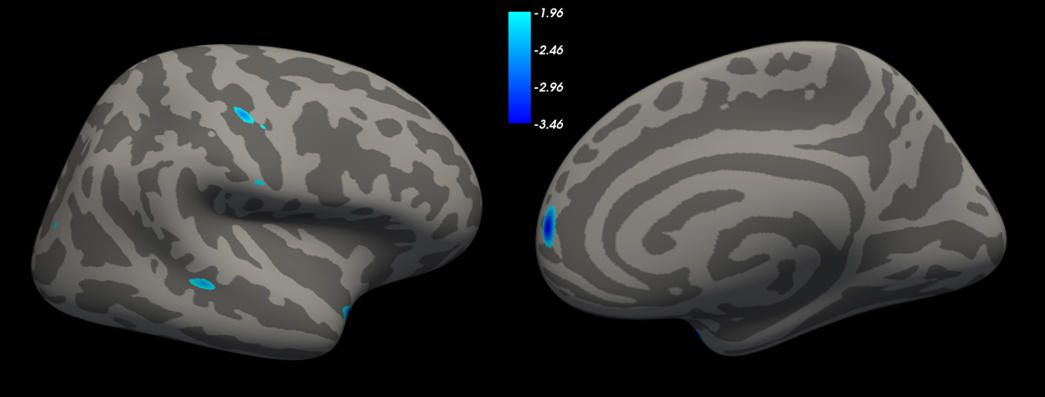


**Supplementary Figure 4.** Clusters with no significant group differences in SA analysis of right cerebrum. Regions with blue color represent the SA of these area was lower in T2DM than health controls, but these are not significantly difficult (vertex *p* < 0.01, cluster *p* > 0.05). Red regions are the opposite.


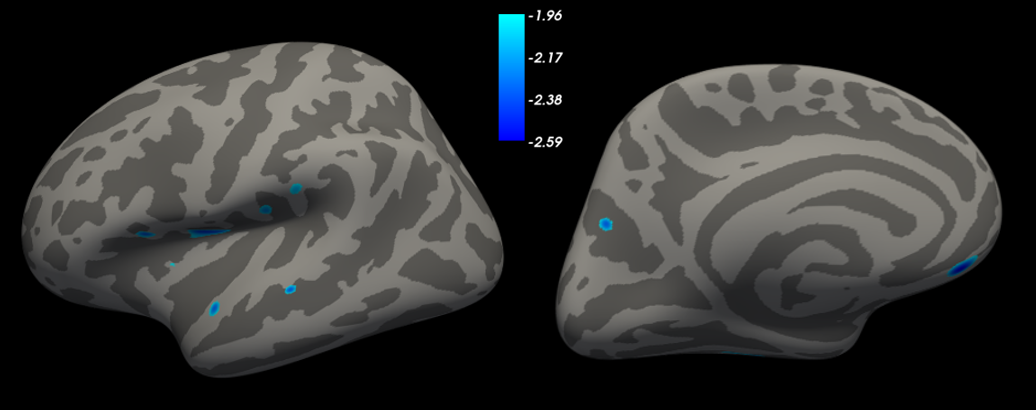


**Supplementary Figure 5.** Clusters with no significant group differences in CV analysis of left cerebrum. Regions with blue color represent the CV of these area was lower in T2DM than health controls, but these are not significantly difficult (vertex *p* < 0.01, cluster *p* > 0.05). Red regions are the opposite.


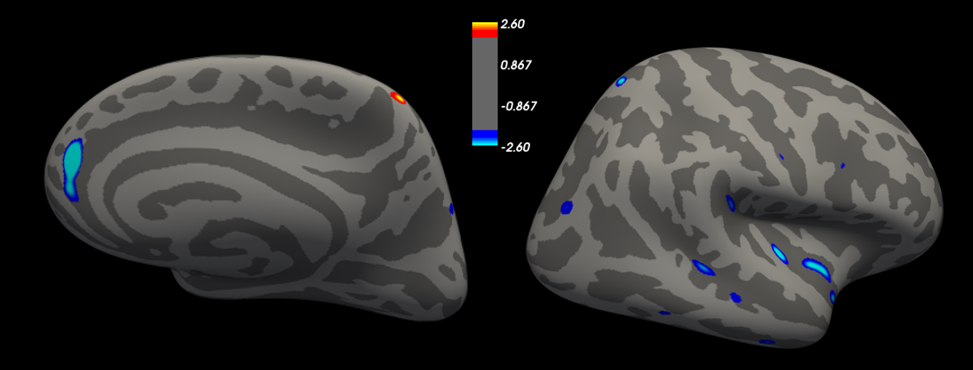


**Supplementary Figure 6.** Clusters with no significant group differences in CV analysis of right cerebrum. Regions with blue color represent the CV of these area was lower in T2DM than health controls, but these are not significantly difficult (vertex *p* < 0.01, cluster *p* > 0.05). Red regions are the opposite.


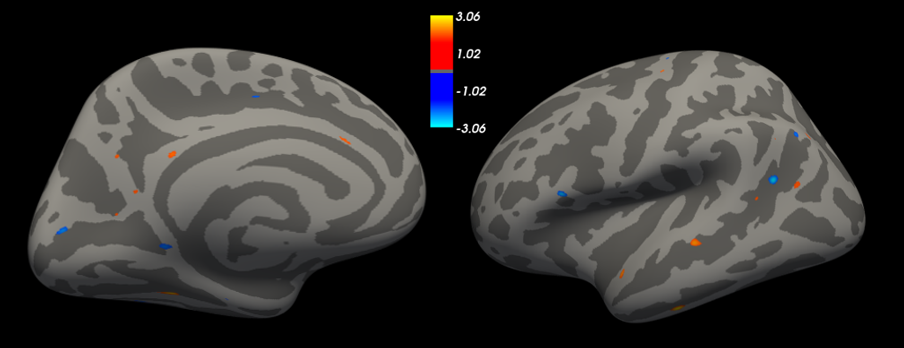


**Supplementary Figure 7.** Clusters with no significant group differences in CSC analysis of left cerebrum. Regions with blue color represent the CSC of these area was lower in T2DM than health controls, but these are not significantly difficult (vertex *p* < 0.01, cluster *p* > 0.05). Red regions are the opposite.


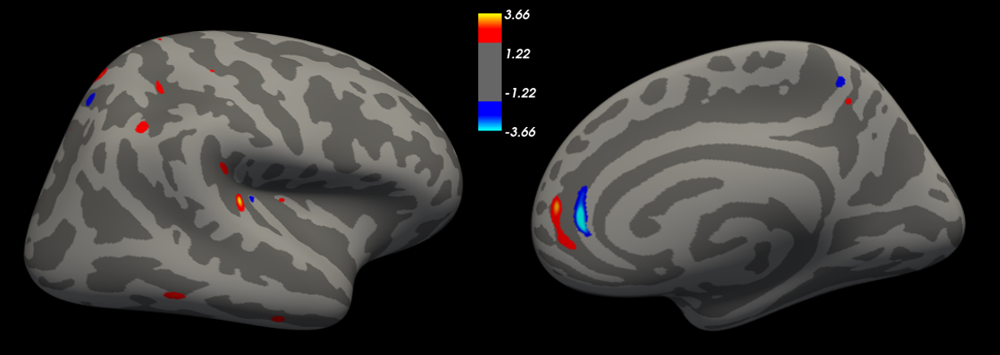


**Supplementary Figure 8.** Clusters with no significant group differences in CSC analysis of right cerebrum. Regions with blue color represent the CSC of these area was lower in T2DM than health controls, but these are not significantly difficult (vertex *p* < 0.01, cluster *p* > 0.05). Red regions are the opposite.
